# Assessment of Nitrogen Ceilings for Dutch Agricultural Soils to Avoid Adverse Environmental Impacts

**DOI:** 10.1100/tsw.2001.333

**Published:** 2001-11-09

**Authors:** Wim de Vries, Hans Kros, Oene Oenema, Jan Willem Erisman

**Affiliations:** Alterra Green World Research, Wageningen, The Netherlands

**Keywords:** ammonia, nitrous oxides, nitrate, eutrophication, biodiversity, nitrogen ceiling, critical loads, modeling, nitrogen balance

## Abstract

In the Netherlands, high traffic density and intensive animal husbandry have led to high emissions of reactive nitrogen (N) into the environment. This leads to a series of environmental impacts, including: (1) nitrate (NO_3_) contamination of drinking water, (2) eutrophication of freshwater lakes, (3) acidification and biodiversity impacts on terrestrial ecosystems, (4) ozone and particle formation affecting human health, and (5) global climate change induced by emissions of N_2_O. Measures to control reactive N emissions were, up to now, directed towards those different environmental themes. Here we summarize the results of a study to analyse the agricultural N problem in the Netherlands in an integrated way, which means that all relevant aspects are taken into account simultaneously. A simple N balance model was developed, representing all crucial processes in the N chain, to calculate acceptable N inputs to the farm (so-called N ceiling) and to the soil surface (application in the field) by feed concentrates, organic manure, fertiliser, deposition, and N fixation. The N ceilings were calculated on the basis of critical limits for NO_3_ concentrations in groundwater, N concentrations in surface water, and ammonia (NH_3_) emission targets related to the protection of biodiversity of natural areas. Results show that in most parts of the Netherlands, except the western and the northern part, the N ceilings are limited by NH_3_ emissions, which are derived from critical N loads for nature areas, rather than limits for both ground- and surface water. On the national scale, the N ceiling ranges between 372 and 858 kton year depending on the choice of critical limits. The current N import is 848 kton year. A decrease of nearly 60% is needed to reach the ceilings that are necessary to protect the environment against all adverse impacts of N pollution from agriculture.

### INTRODUCTION

Human interference in nutrient cycles has increased greatly over the last 2 centuries. Compared to the preindustrial era, human activities (i.e., agriculture, industry, and traffic) have roughly doubled the amount of nitrogen (N) that enters the biosphere[1,2]. Industrialisation and increasing traffic have rapidly increased fossil fuel consumption during the last century, also contributing to increased levels of N in the environment. In various countries in western Europe, nutrient inputs and outputs have even more than doubled as a result of the intensification of the agriculture through increases in fertiliser consumption and animal production, and by industrialisation and increasing traffic[3,4]. On a global scale, the intensification of agricultural production will continue to increase further, as the quest for food and animal protein continues in response to the increasing human population and the increasing prosperity of a great part of the human population[5].

Human interference with the biogeochemical nutrient cycles generally leads to imbalances in nutrient budgets. The recognition that imbalances are not sustainable in the long term has given the impetus to use nutrient budgets as indicators and policy instruments for nutrient management planning. The Netherlands is one of the countries with the highest reactive N emissions density in the world, where reactive N stands for all forms of oxidized and reduced N except for $N_2$. The animal manure production in the Netherlands is approximately five times the average European value per unit of agricultural area[6]. These enhanced levels of reactive N in the environment (in air, soil, ground-, and surface water) lead to a cascade of effects[7]. Observed effects in the Netherlands, for which different targets are defined, include[8]:

1. Pollution of groundwater and drinking water due to nitrate ($NO_3$) leaching (focus on N application and N loss targets);
2. Eutrophication of surface waters, including excess algal growth and a decrease in natural diversity (focus on N application and N loss targets);
3. Decreased species diversity and acidification of non-agricultural soils (focus on $NH_3$ and $NO_x$ ammonia emission targets);
4. Impacts on human health and plants due to ozone for which $NO_x$ is a precursor (focus on $NO_x$ emission targets); and
5. Global warming (focus on $N_2O$ emission targets).

At present, different targets are thus defined in the Netherlands, directed towards different environmental themes. To decrease the leaching of $NO_3$ to groundwater and the runoff of N to surface waters, targets for N losses have been set, where N loss stands for the difference between inputs and outputs at farm level. Annual N loss targets are 60 to 100 kg N ha$^{-1}$ for arable and maize land for fodder production and 140 to 180 kg N ha$^{-1}$ for grassland by the year 2003, depending on the soil drainage status and soil type. These mean N surpluses at farm level have been agreed upon as “satisfactory”[9]. These targets aim to fulfil the EU nitrate directive. The aim is that N leaching and runoff are such that the $NO_3$ concentration in upper groundwater stays below the EU quality criterion of 50 mg l$^{-1}$ and the N concentration in stagnant surface waters below a target concentration of 2.2 mg l$^{-1}$. A Mineral Accounting System (MINAS) has been introduced as a regulatory policy instrument in agriculture to reach the above-mentioned N loss targets and also a phosphate target loss of 20 kg ha$^{-1}$ year$^{-1}$. MINAS is a “regulatory policy instrument” because farmers have to pay sizeable levies when target surpluses have been succeeded. Furthermore, the EU proposed that the maximum animal manure application should be 170 kg ha$^{-1}$ year$^{-1}$.

The original Dutch policy objectives for $NH_3$ emissions were a decrease of 50% by the year 2000 and of 70% by the year 2005, compared to the emissions in the year 1980 of 200 Gg $NH_3$-N[10]. Recently, the Dutch Ministry of Environment has set national $NH_3$ emission targets in Gg $NH_3$ year$^{-1}$ of 100 for the year 2010, 50 for the year 2020, and 30 for the year 2030 to avoid adverse impacts (specifically in view of biodiversity) on natural ecosystems. Note that the $NH_3$ emission targets refer to all $NH_3$ sources, with agriculture being nearly 90% of it. In 1995, annual $NH_3$ emissions in the Netherlands were estimated at 146 Gg $NH_3$-N, equivalent to 175 Gg $NH_3$[11], implying a succeeding decrease in $NH_3$ emissions of approximately 45, 70, and 80% compared to this target year. $NO_x$ emission reductions are also specifically aiming at decreasing N inputs to natural land. The Dutch target is 238 Gg $NO_x$, whereas the present emissions are approximately 370 Gg $NO_x$. Finally, the emission target for $N_2O$ is a 6% decrease compared to 1995, whereas the ultimate target is background emission.

On the basis of the above-mentioned targets, several measures have been implemented in Dutch agriculture to control reactive N emissions. It appears that these measures are not as effective as predicted beforehand, either because its effectiveness is lower than expected or it is compensated by growth of the activity[8]. Furthermore, it was found that measures taken to decrease emissions have led to a shift in other emissions. Examples of this are the prohibition to apply slurry in winter to decrease leaching of $NO_3$, which induced higher $NH_3$ emissions because slurry was applied at higher temperatures[12]. The example given above illustrates the need for an integrated N approach, since measures to decrease N leaching negatively affects N impacts due to eutrophication and acidification of natural ecosystems. This has led to discussion about the possibilities to develop a more successful N policy.

To allow the implementation of an integrated N policy, we calculated “regional specific ceilings for reactive N”. The N ceiling is defined here as the maximum amount of reactive N that does not lead to exceedance of critical limits or targets, which aim to protect the environment, within or outside the region. Examples are critical limits for ambient concentrations of reactive N in the atmosphere or targets for N emissions in view of such limits ($NH_3$, $NO_x$, and $N_2O$ emission targets), critical deposition loads of reactive N to sensitive areas, and critical concentrations of $NO_3$ or total N in ground- and surface water. In this paper, we present results of calculated N ceilings for various criteria with respect to environmental protection, using a simple model for the N balance in terrestrial (agricultural) and aquatic ecosystems on a national scale. Criteria are limited to targets for $NH_3$ emission, $NO_3$ concentrations in groundwater, and total N concentrations in surface water, since those protection goals are most limiting the acceptable N input in agriculture. If those criteria are met, all others are met as well[8].

### MODELLING APPROACH AND MODEL APPLICATION

#### Modelling Approach

To gain insight into the N ceilings for agricultural soils in the Netherlands, a model was developed called INITIATOR (Integrated NITrogen Impact AssessmenT model On a Regional scale), representing all crucial processes in the N chain by simple process descriptions[13]. A flow chart of the considered N inputs and N transformation processes in the model for both terrestrial and aquatic ecosystems is given in Fig. 1. We have chosen a simple approach to maintain transparency and to be able to apply the model with available data.

INITIATOR is a simple N balance model based on empirical linear relationships between the different N fluxes. In agricultural systems, first the total N input to the soil is calculated as the sum of inputs via animal manure, fertiliser, atmospheric deposition, and biological N fixation. The fate of N in the terrestrial system is calculated as a sequence of occurrences in the order $NH_3$ emissions, followed by N uptake, N accumulation/immobilisation, nitrification, and denitrification in the soil. All N transformation processes are linearly related to the inflow of N (first order kinetics). This implies that $NH_3$ emission due to application depends linearly on the N input into the soil, N uptake on the N input minus the $NH_3$ emission, N immobilisation on the input minus the $NH_3$ emission minus N uptake, etc. The linear transformation constants are a function of type of manure, land use, soil type, and/or hydrological regime. The parameterisation of the equations for estimating the $NH_3$ loss was done in such a way that it included all $NH_3$ losses, including those from animal housing and manure storage systems and from the application of animal manure, fertilisers, and dung and urine from grazing animals to the soil. In the approach it was implicitly assumed that the manure that was applied to the soil in a given grid cell (external data) came from the farms in the same grid cell.

The flux of N leaving the terrestrial system is calculated by subtracting all N outputs from the system (emission, uptake, net accumulation, and denitrification) from the N inputs to the soil. The leaching loss from the terrestrial systems is partitioned to surface water and to groundwater by multiplying the leaching loss with a runoff fraction (including all pathways for N moving to surface waters) and a leaching fraction (1 - runoff fraction), respectively. Since we are interested in the leaching of N to the groundwater at 1-m depth below the phreatic level (the depth where $NO_3$ concentrations are measured in the Netherlands), denitrification of N in upper groundwater is also considered. The processes considered relevant in aquatic systems are N retention in ditches and larger surface waters, retention being distinguished in denitrification and accumulation in the sediment. Denitrification is thus calculated in the soil, upper groundwater, ditches, and surface water (compare Fig. 1). We considered runoff from terrestrial systems and direct atmospheric deposition of N to surface waters as input of N in aquatic systems. The various N outputs from - and the N immobilisation in soil, groundwater, and surface water are calculated with a consistent set of simple linear equations[13].

### Calculation of N Ceilings

The unique point of INITIATOR is that the model is also able to calculate an acceptable N input to the soil or N ceiling in agriculture on the basis of different quality criteria. This includes a critical limit for the $NO_3$ concentration in upper groundwater

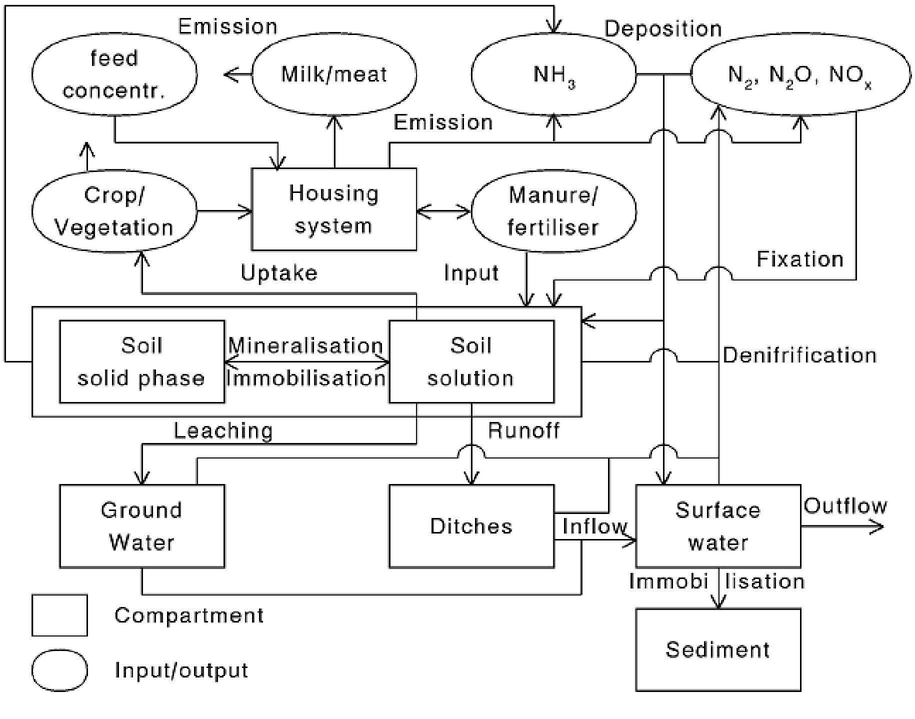

**FIGURE 1.** Overview of the N inputs and N processes in terrestrial and aquatic ecosystems considered in INITIATOR.

(50 mg $l^{-1}$), for the N concentration in stagnant surface water (2.2 mg $l^{-1}$) and $NH_3$ emission targets[8]. The acceptable N input to the soil equals the N inputs in animal manure, fertiliser, deposition, and biological N fixation, whereas N ceilings on farm level are equal to the acceptable N inputs via animal feed concentrates, fertiliser, deposition, and biological N fixation.

As stated before, the model calculates N leaching and runoff from the N input minus $NH_3$ emission, net N removal through agricultural products, N immobilisation, and denitrification in both soil and surface water. Since all N loss fluxes from the system are described sequentially, the model can derive those fluxes also for a calculated critical N leaching rate to groundwater and N runoff rate to surface water. Those critical N fluxes can be derived by multiplying leaching rate to groundwater with the target of 50 m $l^{-1}$ for the $NO_3$ concentration or the runoff rate to surface water with the target of 2.2 mg $l^{-1}$ for the N concentration. In calculating the acceptable N input, the corresponding $NH_3$ emission is also calculated, assuming that the ratio between the N input by animal manure (divided in cattle manure, pig manure, and poultry manure, and dung/urine from grazing animals) and fertilisers stays the same as in the year 2000. By comparing this emission with the acceptable $NH_3$ emission related to the protection of biodiversity of natural areas, the maximum acceptable N input can be calculated such that the biodiversity of natural areas, groundwater quality, and surface water quality are all protected.

The acceptable $NH_3$ emission at each plot was calculated for the Netherlands on the basis of available data for critical N loads related to impacts of N deposition on biodiversity, nutrient imbalances, and groundwater quality[14] using emission-deposition relationships in combination with an optimisation procedure. The approach is illustrated in Fig. 2[8]. The acceptable N input is recalculated when the acceptable $NH_3$ emission related to protection of biodiversity of natural areas is lower than the $NH_3$ emission related to protection of groundwater and surface water quality. In this case, the fertiliser input is kept constant, since decrease of fertiliser input is not adequate in decreasing $NH_3$ emissions. Instead, the input by animal manure is scaled back, assuming that the ratio between the N input by cattle manure, pig manure, poultry manure, and dung/urine from grazing animals stays the same as in the year 2000.

### Model Application

The modelling approach was applied to the whole of the Netherlands, distinguishing grid cells with unique combinations of soil type, land use, N inputs, and hydrology, which determine the

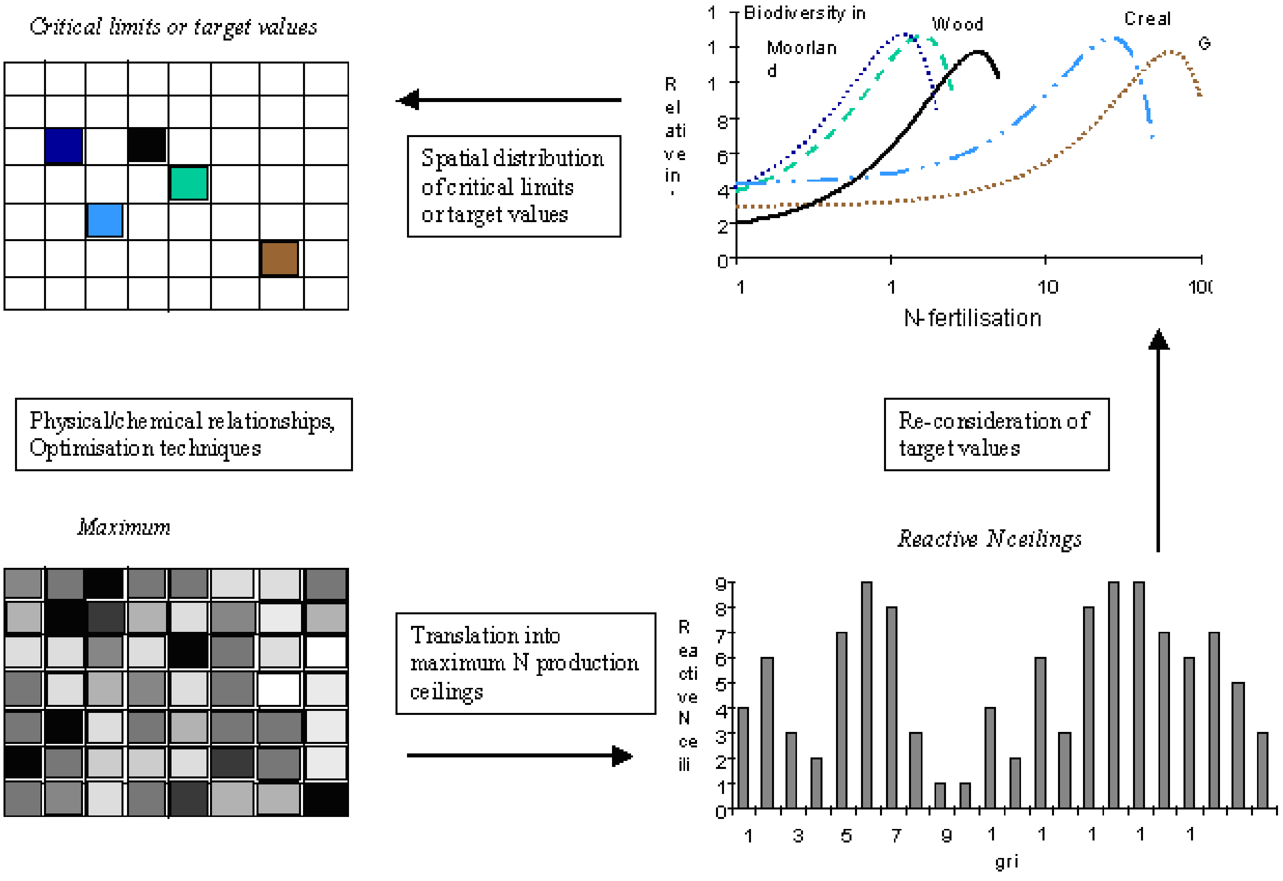

**FIGURE 2.** Illustration of the calculation of reactive N ceilings on a regional scale parameterisation of N transformation processes. For agriculture, a total number of 2543 plots were distinguished, consisting of a multiple of 500- × 500-$m^2$ grid cells with unique combinations of soil use, soil type, and groundwater table class[15]. Georeferenced data for the N input via animal manure and fertilisers were based on data statistics at farm and municipal level for the year 2000, using the CLEAN model[16]. Animal manure was divided in cattle, pig, and poultry manure, and in dung and urine deposited on grassland by grazing animals, since this has an influence on the $NH_3$ emissions from either housing and manure storage systems or from the soil. N deposition data were based on modelled N deposition data at a 1- × 1-km grid scale for the year 2000, using the model DEADM[12]. N fixation was estimated as a function of land use[13].

The model parameters describing N transformation processes and transfers were estimated as a function of land use, soil type, and groundwater table class, thus allocating them to combinations occurring in distinct plots. In the agricultural plots, a distinction was made between grassland, maize, and arable land. Soils were divided in sand, loess, clay, and peat. Furthermore, a distinction was made between different hydrological regimes (wetness classes), using groundwater table classes (Gt) from a 1:50,000 soil map with information on the mean highest water level (MHW) used in the plots, according to: (1) wet (poorly drained): MHW <40 cm; (2) moist (moderately drained): MHW 40 to 80 cm; and (3) dry (well drained): MHW >80 cm. Model parameters describing the various N transformations were based on literature data, field observations, results from more detailed model calculations, and expert judgement[13].

In this study, the $NH_3$ emission fractions from animal housing systems were assumed equal to those from low emission (green lable) stables. This is foreseen for all Dutch farms in the future and is needed when one wants to reach the $NH_3$ emission targets. For reasons of comparison, this effect was included in all the calculations of N ceilings, including when ground- and surface water only were protected. Furthermore, in situations where the $NH_3$ emissions produced by cows eating grass and maize only (no feed concentrates) already exceeds the acceptable N input, we assumed that part of the animal manure produced is exported. This is needed to still have animal husbandry and reach the targets. Otherwise, the land has to be abandoned.

### MODEL RESULTS

Model results include: (1) N fluxes to air, ground-, and surface water, (2) N ceilings and acceptable soil inputs, and (3) areas exceeding different N ceilings when implementing the Mineral Accounting System (MINAS) and the EU target of 170 kg $ha^{-1}$ $year^{-1}$ of animal manure, as described in more detail below.

#### N Fluxes to Air, Ground-, and Surface Water for Different Protection Levels

National estimates of N fluxes to air, ground-, and surface water for various criteria with respect to the protection of groundwater, surface water, and nature areas are given in Table 1. Estimated total N inputs to agricultural farms were slightly lower for animal feed concentrates (362 Gg N $year^{-1}$) than for fertiliser (396 Gg N $year^{-1}$). N deposition was estimated at 49 Gg N $year^{-1}$ and biological fixation at 41 Gg N $year^{-1}$, leading to a total

**TABLE 1**
**N Ceilings, Acceptable Soil N Input, and N Fluxes to Air, Groundwater, and Surface Water Related to the Protection of Various Combinations of Groundwater, Surface Water, and Nature in View of Optimised $NH_3$ Ceilings of 93 and 50 Gg $NH_3$**

| Environmental Protection Goal | N ceiling (Gg N $year^{-1}$) | Acceptable N Input Soil (Gg N $year^{-1}$) | $NH_3$ Emission (Gg $NH_3$ $year^{-1}$) | $N_2O$ Emission (Gg N $year^{-1}$) | N Inflow to Groundwater (Gg N $year^{-1}$) | N Inflow to Surface Water (Gg N $year^{-1}$) |
|---|---|---|---|---|---|---|
| Input in 2000 | 848 | 950 | 68 (160)[a] | 29 | 50 | 15 |
| GW + P-limit[b] | 858 | 955 | 108 | 38 | 37 | 11 |
| GW + SW[b] | 646 | 776 | 79 | 33 | 11 | 5 |
| GW + AM93[b] | 630 | 784 | 71 | 29 | 26 | 10 |
| GW + AM50[b] | 504 | 618 | 42 | 20 | 18 | 7 |
| GW + SW + AM93 | 436 | 560 | 49 | 19 | 10 | 4 |
| GW + SW + AM50 | 372 | 466 | 32 | 14 | 7 | 3 |

[a] The value in brackets is the calculated $NH_3$ emission at present without conversion to low emission stables. The value of 160 Gg $NH_3$ $year^{-1}$ is equal to a national average emission of 132 Gg N $year^{-1}$.

[b] GW stands for protection of upper *groundwater* ($NO_3$ concentration stays below the EU quality criterion of 50 mg $l^{-1}$), SW for protection of stagnant *surface waters* (N concentration stays below a target concentration of 2.2 mg $l^{-1}$), and AM93 and AM50 for annual $NH_3$ ceilings of 93 and 50 Gg $NH_3$ to protect biodiversity in nonagricultural areas.

input of 848 Gg N year$^{-1}$. The animal manure produced from both feed concentrates and forage was, however, slightly higher than the fertiliser input (464 Gg N year$^{-1}$). About 66% of this amount originated from cattle, 23% from pigs, and 11% from poultry, including other animals. The total N input to the soil, equal to the sum of fertiliser, animal manure, deposition, and fixation, was calculated at 950 Gg N year$^{-1}$.

Using the input for the year 2000, annual fluxes (Gg N year$^{-1}$) of 132 for $NH_3$ emission (160 Gg $NH_3$ year$^{-1}$), 29 for nitrous oxide emission, 50 for N inflow to groundwater, and 15 for N inflow to surface water were calculated. The N inflow to ground- and surface water has to be decreased by approximately 25% when the N ceiling related to groundwater protection has to be reached, and by nearly 80 to 70%, respectively, when N ceiling related to both ground- and surface water protection has to be reached. The need to reach $NH_3$ emission targets in addition to groundwater protection still causes a clear decrease in N inflow to both ground- and surface water. The additional gain is small when $NH_3$ emission targets need to be reached in addition to ground- and surface water protection (Table 1).

The present $NH_3$ emission decreases from an estimated value of 160 to 68 Gg $NH_3$ year$^{-1}$ when all housing systems are converted to low emission systems. This amount was calculated to increase (108 Gg $NH_3$ year$^{-1}$) when one would only protect groundwater, due to the calculated possible increase in manure application instead of fertilisers (see Table 3). The $NH_3$ emission related to the protection of ground- and surface water is slightly higher (79 Gg $NH_3$ year$^{-1}$) than the $NH_3$ emission related to groundwater protection only and an emission target of 93 Gg $NH_3$ year$^{-1}$ (71 Gg $NH_3$ year$^{-1}$), despite the lower N ceiling in the first case. This is again because the ratio between fertiliser and animal manure is different in both cases (Table 3). When one only focuses on leaching to ground- and surface water, it is relatively efficient to decrease fertiliser inputs, whereas the decrease of $NH_3$ emissions is best obtained by decreasing animal manure inputs. The calculated $NH_3$ emission of 71 Gg $NH_3$ year$^{-1}$ is lower than the emission target of 93 Gg $NH_3$ year$^{-1}$, since groundwater protection is sometimes more stringent than this $NH_3$ emission target. This is even more true for surface water. For example, the combination of ground- and surface water protection and an emission target of 93 Gg $NH_3$ year$^{-1}$ leads to an emission of 49 Gg $NH_3$ year$^{-1}$ only.

The decrease of the N input in agriculture will lead to a significant decrease in the emission of the greenhouse gas $N_2O$. At present, the aim for $N_2O$ is a 6% decrease compared to the year 1995. The results show that if the $NH_3$ targets would be reached in combination with the protection of ground- and surface water, the decrease in $N_2O$ emission is likely to be much larger. This implies that the target for $N_2O$ emission decrease is less ambitious than the targets for $NH_3$. The calculated increase in $N_2O$ emission for the targets related to groundwater only, or even ground- and surface water, compared to the present situation is due to the different allocation of manure and fertiliser to the land. To meet the targets, it is efficient to spread the manure preferentially on peat and clay soils with a high denitrification potential. This does, however, cause an increase in $N_2O$ emission, which is related to denitrification.

### N Ceilings and Acceptable Soil Inputs for Different Protection Levels

National estimates obtained for N ceilings on farm level (input by feed concentrates, fertiliser, deposition, and biological fixation) were always lower than the acceptable N application to the soil (input by animal manure, fertiliser, deposition, and biologi-

**TABLE 2**
**N Ceilings Related to the Protection of Various Combinations of Groundwater, Surface Water, and Optimised $NH_3$ Ceilings, Including and Excluding Export of Manure**

| Environmental Protection Goal | N Ceiling with Manure Export (Gg N year$^{-1}$) | | | N Ceiling without Manure Export (Gg N year$^{-1}$) | | |
|---|---|---|---|---|---|---|
| | Feed Concentrates | Fertiliser | Total[a] | Feed Concentrates | Fertilizer | Total[b] |
| Input in 2000 | 362 | 396 | 848 | 362 | 396 | 848 |
| GW + P-limit[b] | 456 | 312 | 858 | 454 | 310 | 854 |
| GW + SW[b] | 330 | 226 | 646 | 295 | 164 | 549 |
| GW + AM93[b] | 211 | 329 | 630 | 195 | 262 | 547 |
| GW + AM50[b] | 89 | 325 | 504 | 81 | 109 | 280 |
| GW + SW + AM93 | 120 | 226 | 436 | 84 | 150 | 323 |
| GW + SW + AM50 | 58 | 224 | 372 | 31 | 56 | 177 |

[a] Total N ceiling includes the input by feed concentrates, fertiliser, deposition, and biological fixation. The latter two inputs were fixed at the input of 90 Gg N year$^{-1}$ in 2000, i.e., 49 Gg N year$^{-1}$ for deposition and 41 Gg N year$^{-1}$ for biological fixation.

[b] GW stands for protection of upper *groundwater* ($NO_3$ concentration stays below the EU quality criterion of 50 mg l$^{-1}$), SW for protection of stagnant *surface waters* (N concentration stays below a target concentration of 2.2 mg l$^{-1}$), and AM93 and AM50 for annual $NH_3$ ceilings of 93 and 50 Gg $NH_3$ to protect biodiversity in nonagricultural areas.

**TABLE 3**
**Acceptable Soil N Inputs Related to Different Criteria for Groundwater, Surface Water, and Optimised $NH_3$ Ceilings, Including and Excluding Export of Manure**

| Environmental Protection Goal | Allowable N Input to Soil with Manure Export (Gg N year$^{-1}$) | | | | Allowable N Input to Soil without Manure Export (Gg N year$^{-1}$) | | |
|---|---|---|---|---|---|---|---|
| | Manure | Fertiliser | Export | Total[a] | Manure | Fertiliser | Total[a] |
| Input in 2000 | 464 | 396 | — | 950 | 464 | 396 | 950 |
| GW + P-limit[b] | 552 | 312 | 1 | 955 | 550 | 310 | 950 |
| GW + SW[b] | 409 | 226 | 51 | 776 | 348 | 164 | 602 |
| GW + AM93[b] | 335 | 329 | 30 | 784 | 334 | 262 | 686 |
| GW + AM50[b] | 96 | 325 | 107 | 618 | 144 | 109 | 343 |
| GW + SW + AM93 | 167 | 226 | 57 | 560 | 177 | 150 | 417 |
| GW + SW + AM50 | 41 | 224 | 109 | 466 | 70 | 56 | 216 |

[a] Total N input to the soil includes the input by animal manure applied to the land, fertiliser, deposition, and biological fixation, correcting for export. The latter two inputs were fixed at the input of 90 Gg N year$^{-1}$ in 2000.

[b] GW stands for protection of upper *groundwater* ($NO_3$ concentration stays below the EU quality criterion of 50 mg l$^{-1}$), SW for protection of stagnant *surface waters* (N concentration stays below a target concentration of 2.2 mg l$^{-1}$), and AM93 and AM50 for annual $NH_3$ ceilings of 93 and 50 Gg $NH_3$ to protect biodiversity in nonagricultural areas.

cal fixation) as indicated in Table 1. Apparently, the $NH_3$ emission from the animal housing systems, included in the calculation of the N ceiling, is less than the recycling of N in manure due to intake of forage (grass and maize), which is included in the acceptable N application to the soil.

The division of N ceilings and acceptable N inputs to soil in the various sources are given in Tables 2 and 3, respectively. On a national scale, the N ceiling and the acceptable N input at the soil surface is slightly higher than the present input, when one wants to protect groundwater only. Protection of groundwater can thus in principle be reached by a redistribution of the animal manure and fertiliser application over the Netherlands. Results show that a lower input of fertilisers (see modeling approach) leads to more animal manure input (Table 3), but this does increase the $NH_3$ emission (Table 1). The national N ceiling related to the protection of both ground- and surface water is almost comparable to the N ceiling related to the protection of groundwater only and an $NH_3$ emission target of 93 Gg $NH_3$ year$^{-1}$ (Table 2). To a slightly lesser extent, this is also true for the soil N input (Table 3). In most parts of the Netherlands, the $NH_3$ emission targets are, however, more restrictive than those based on limits for groundwater, especially when the target of 50 Gg $NH_3$ year$^{-1}$ is used. The critical limit for surface water is nearly always more stringent than for groundwater, but also here the $NH_3$ emission targets are often more restrictive, especially when the target of 50 Gg $NH_3$ year$^{-1}$ is used (Table 2).

The most stringent approach (protection of ground- and surface water and an $NH_3$ emission target of 50 Gg $NH_3$ year$^{-1}$) leads to a national acceptable N input to the soil of 466 Gg year$^{-1}$ and an N ceiling of 372 Gg year$^{-1}$. This is, however, based on the assumption of a net export of animal manure or animal manure processing at the farm (Table 3). This is needed at those plots where the amount of N in animal manure produced at zero import of animal feed concentrates leads to N emissions to air or water exceeding the acceptable limits. Compared to the current N ceiling of 848 Gg year$^{-1}$, a decrease of nearly 60% is needed to reach this N ceiling considered necessary to protect the environment against all adverse impacts resulting from N input to agriculture. Assuming that the fertiliser input will be decreased less drastically, this implies that the N input by feed concentrates has to be decreased by more than 80% in this case (Table 2). The necessary decrease in animal manure input, assuming manure export or processing and low emission housing, is nearly 70% (from 464 to 150 Gg N year$^{-1}$, being the sum of 41 and 109 Gg N year$^{-1}$; Table 3).

Especially in the situation where stringent $NH_3$ targets have to be reached, it is impossible to still have animal husbandry at certain plots unless manure is exported or processed at the farm. Otherwise, the land has to be abandoned, causing zero input by feed concentrates, animal manure, fertiliser, and negligible $NH_3$ emission. Calculations of the N ceiling and acceptable soil N input thus obtained are presented in the right-hand side of Tables 2 and 3. In this calculation, we assumed that the N input by deposition and fixation is taken up by natural grassland, leading to negligible leaching and runoff. Results thus obtained lead to lower soil N inputs and N ceilings. The amount of manure that is acceptable is lower than the sum of manure application and manure export (or processing) in the first case (see left-hand side of Table 3). The acceptable rate of animal manure application to the land is, however, higher for the criteria including $NH_3$ emission targets. This is because the amount of fertiliser that is applied decreases strongly due to land abandonment. The area that has to be set aside is calculated to increase from 20,825 ha when protecting groundwater only to 1.498825 ha when protecting

**TABLE 4**
**Areas Exceeding Different N Ceilings when Implementing the Mineral Accounting System (MINAS) and the EU Target of 170 kg ha$^{-1}$ year$^{-1}$ for Animal Manure Application**

| Environmental Protection Goal | Area Exceeding N Ceilings (%) | | |
|---|---|---|---|
| | Present | MINAS | EU |
| GW + P-limit[a] | 57 | 22 | 28 |
| GW + SW[a] | 83 | 77 | 78 |
| GW + AM93[a] | 81 | 66 | 77 |
| GW + AM50[a] | 92 | 79 | 88 |
| GW + SW + AM93 | 95 | 84 | 87 |
| GW + SW + AM50 | 98 | 94 | 97 |

[a] GW stands for protection of upper *groundwater* ($NO_3$ concentration stays below the EU quality criterion of 50 mg l-1), SW for protection of stagnant *surface waters* (N concentration stays below a target concentration of 2.2 mg l-1), and AM93 and AM50 for annual $NH_3$ ceilings of 93 and 50 Gg $NH_3$ to protect biodiversity in nonagricultural areas.

groundwater, surface water, and nature. This is approximately 1 to 70% of the agricultural area of the Netherlands. For the other options, it varies between 15% (groundwater protection and an emission target of 93 $NH_3$ year$^{-1}$) and 60% (groundwater protection and an emission target of 50 $NH_3$ year$^{-1}$).

### The Impacts of the Present Manure Application Targets on the Protection Level of Groundwater, Surface Water, and Nature

Despite the relative low N input to ground- and surface water compared to the N input to soil (about 6 and 1.5%, respectively), it does cause an exceedance of critical limits for $NO_3$ in groundwater (50 mg l$^{-1}$) and N in surface water (2.2 mg l$^{-1}$) in large parts of the Netherlands at present. This is illustrated in Table 4, which shows that the N ceiling based on either the target of 50 mg l$^{-1}$ for the $NO_3$ concentration in groundwater or a phosphate target loss of 20 kg ha$^{-1}$ year$^{-1}$ is presently exceeded at 57% of the plots. The latter target was included to avoid extremely high acceptable N inputs for clay and peat soils with a very high denitrification potential. The calculated area exceeding a critical $NO_3$ limit of 50 mg l$^{-1}$ alone was only 26%, but the area exceeding the targets for both groundwater and surface water is 83%.

We calculated that application of the MINAS system does indeed decrease the area exceeding the critical limit for $NO_3$ of 50 mg l$^{-1}$ and the phosphate target loss of 20 kg ha$^{-1}$ year$^{-1}$ for groundwater considerably (from 57 to 22%; Table 4). This holds specifically for the phosphate target loss, however, since the calculated area exceeding a critical $NO_3$ limit of 50 mg l$^{-1}$ decreased from 26 to 21% only. Exceedances are thus still to be expected with MINAS, especially in dry sandy soils and in loess soils on grassland with an N loss target of 140 kg ha$^{-1}$ year$^{-1}$. MINAS, however, aims to decrease target N surpluses to such a level that not only $NO_3$ concentrations in upper groundwater but also N concentrations in surface waters do not exceed target concentrations. Considering the N ceiling for both ground- and surface water, the impact of MINAS on the area exceeding the critical limit of 2.2 mg l$^{-1}$ for surface water is calculated to be much less (from 83 to 77%). Finally, the decrease in N inputs by MINAS do cause a decrease in $NH_3$ emissions from approximately 160 to 115 Gg $NH_3$ year$^{-1}$, but the area exceeding optimised $NH_3$ emission targets of 93 and especially 50 Gg $NH_3$ year$^{-1}$, set by the Dutch Government, hardly decrease (Table 4).

## DISCUSSION AND CONCLUSIONS

### Results in View of Other Studies

This paper describes results of an integrated analysis using the newly developed INITIATOR model. INITIATOR has not yet been thoroughly validated, but calculated national averages of those N fluxes, i.e., $NH_3$ emission, $N_2O$ emission, and total leaching to ground- and surface water, are in line with most previous studies using more sophisticated models[11,17]. Results for the year 1995 are within 5% for most of those fluxes[13]. A major difference with previous studies is that they do mostly focus on parts of the system (for example, on either $NH_3$ emission or $N_2O$ emission to the atmosphere or leaching to groundwater or runoff to surface waters), but hardly ever on the overall fate of N.

More importantly, another major difference is the possibility of calculating “reactive N production ceilings” for agriculture. This paper presents a preliminary quantification of N ceilings for Dutch agricultural soils on a national scale. It is shown that it is possible to set limits to N inputs by fertilisers and feed concentrates on a regional scale to meet the environmental limits.

### Model Results

The first calculations show that the reactive N import in the Netherlands has to be decreased by 60% and regionally optimised in order to reach the environmental limits. Regionally there are large differences between the decreases needed. There are a few areas in the Netherlands where, according to this preliminary analysis, no decreases are needed, but especially in the intensive agriculture areas in the centre, east, and south of the country, a strong decrease in N input is needed. This calculation is, however, based on the assumption that at each plot, the locally optimised $NH_3$ emission target has to be reached. Due to this approach, the $NH_3$ emission is always lower than the given national target. The most stringent approach (protection of ground- and surface water and an $NH_3$ emission target of 50 Gg $NH_3$ year$^{-1}$) leads to an actual emission of approximately 30 Gg $NH_3$ year$^{-1}$, being the Dutch target for the year 2030. Another approach is to give such a target for a larger area (all farms in a certain area should not exceed an $NH_3$ emission target) without optimising the $NH_3$ emission targets within the area. In general, the choice of critical limits or target values used in these calculations, which limit the production of reactive N and the associated activities, is very important. The model results should be considered with care since an uncertainty of approximately 25 to 50% is likely is most model parameters[13]. Nevertheless, it shows the directions that are needed in Dutch agriculture when certain targets are to be reached.

### Policy Implications

Maximum N ceilings based on all critical limits (nature, groundwater, surface water, greenhouse gas emission, etc.) provide a solid basis for an integrated N policy. It limits all the N related impacts at the same time, prevents shifts to other environmental issues, and is therefore efficient, cost effective, and probably more acceptable to the target groups. An important aspect of a successful integrated N policy is also related to the possibilities for inspection and maintenance of implementation[8]. This aspect has not been addressed here. A N trading system might be set up which stimulates the implementation of measures to reach the ceilings, comparable to the Mineral Accounting System (MINAS). Emission trades are also possible between different target groups. A N accounting system in which the N balance is calculated regionally and locally (individual farms) using standard/prescribed methods can assist such a system. The excess N might be taxed to stimulate decreases. The system can give guidance to the spatial planning of activities and room for expansion in different areas, and it can stimulate technological developments in the right direction.

Measures to reach N ceilings should be focused on decreasing the production of reactive N and/or the import of it. Solutions to reach the N production ceilings might be sought in technological measures aimed at conversion of reactive N into $N_2$, preventing reactive N from being formed, or limiting reactive N import. This approach is not only useful for the Netherlands, with its excess N inputs, but also for other countries in the world where either N becomes a threat or need limits to growth in order to prevent situations of unbalanced N.

| | | |
|---|---|---|
| Received: | August | 8, 2001 |
| Revised: | October | 12, 2001 |
| Accepted: | October | 29, 2001 |
| Published: | November | 9, 2001 |

### BIOSKETCH

**Dr. Wim de Vries** is Principal Research Scientist and Research Programme Leader in the field of soil chemistry at Alterra Green World Research, Wageningen, the Netherlands, specializing in soil acidification, nutrient cycling, carbon sequestration, and heavy metal pollution. His focus is the assessment critical loads for nitrogen, sulphur and heavy metals on terrestial ecosystems. Dr. de Vries received his Ph.D. degree (cum laude, with honour) from the Agricultural University Wageningen, the Netherlands. His doctoral thesis focussed on soil response to acid deposition at different regional scales. Current research interests include the development and application of soil models at various regional scales (local, regional, transnational) in combination with field and laboratory research, and effects on air pollution on terrestrial ecosystems, with special reference to forests.
